# *Streptococcus suis *in Brazil: Genotypic, Virulence, and Resistance Profiling of Strains Isolated from Pigs between 2001 and 2016

**DOI:** 10.3390/pathogens9010031

**Published:** 2019-12-28

**Authors:** Carlos E. C. Matajira, Luisa Z. Moreno, Andre P. Poor, Vasco T. M. Gomes, Andressa C. Dalmutt, Beatriz M. Parra, Carolina H. de Oliveira, Mikaela R. F. Barbosa, Maria Inês Z. Sato, Franco F. Calderaro, Andrea M. Moreno

**Affiliations:** 1 Departamento de Medicina Veterinária Preventiva e Saúde Animal, Faculdade de Medicina Veterinária e Zootecnia, Universidade de São Paulo, São Paulo 01000-000, Brazil; k.rlos89.cabrera@gmail.com (C.E.C.M.); luzanolli@gmail.com (L.Z.M.); andrepegoraro21@gmail.com (A.P.P.); vascotulio@usp.br (V.T.M.G.); adalmutt@usp.br (A.C.D.); bmartinsparra@gmail.com (B.M.P.); ch-oliveira@outlook.com.br (C.H.d.O.); 2 Companhia Ambiental do Estado de São Paulo (CETESB), São Paulo 01000-000, Brazil; mrbarbosa@sp.gov.br (M.R.F.B.); misato@sp.gov.br (M.I.Z.S.); 3 Universidade Paulista-UNIP, São Paulo 01000-000, Brazil; calderarofranco@hotmail.com

**Keywords:** *Streptococcus suis*, serotyping, antimicrobial resistance, PFGE, Brazil

## Abstract

*Streptococcus suis* remains an important challenge for the worldwide swine industry. Considering that Brazil is a major pork producer and exporter, proper monitoring of the pathogen and resistance rates are required. We present here the characterization of Brazilian *S. suis* strains isolated over a 15 year period by pulsed-field gel electrophoresis (PFGE) typing, capsular, virulence, and antimicrobial resistance profiling. Serotype prevalence revealed a predominance of serotype 2/½ followed by 3, 7, 1/14, 6, 8, 18, 28, and 27; the latter had not yet been reported in Brazil. Resistance profiling enabled the differentiation of nine profiles presenting resistance to three and up to eight antimicrobial classes. Even though an association between the most resistant strains and isolation year starting from 2009 was observed, a high frequency of multidrug-resistant strains isolated from 2001 to 2003 was also detected. This suggests that despite the isolation period, *S. suis* strains already presented high resistance selection pressure. A slight association of serotype 2/½ with some virulence profiles and PFGE pulsotypes was also identified. Nevertheless, no clonal dispersion or persistency of clones over the analyzed years and herds was detected.

## 1. Introduction

Despite constant efforts to control the occurrence of *Streptococcus suis* infections in swine herds and the use of vaccines, this pathogen remains an important challenge for the swine industry in Brazil and worldwide. In swine, this species causes meningitis, arthritis, endocarditis, septicemia, polyserositis, pneumonia and sudden death, and the control and prevention of *S. suis* infections require constant investments [1].

This bacterium also presents significant zoonotic potential, being described in different countries as a cause of deafness, meningitis, arthritis, and septicemia in people who work in swine herds, slaughterhouses or butchers, and meat consumers [2,3]. In Brazil, to date, there are no reports of human infection by *S. suis* [4].

Based on its capsular polysaccharide antigens, *S. suis* was classified into 35 serotypes [5], of which serotype 2 is the most common in diseased animals and humans [1,2]. However, recent studies have shown that serotypes 20, 22, 26, 32, 33, and 34 do not belong to this species and should be classified as other bacterial species [6,7]. Moreover, novel nine capsular polysaccharide synthesis (*cps*) loci (NCLs) of non-typable *S. suis* strains have been identified based on DNA sequencing. Therefore, strict *S. suis* species currently comprise 38 serotypes [8].

Among the virulence factors that have already been characterized in *S. suis, *most studied so far are the capsule, muramidase-released protein (MRP), the extracellular factor (EF), hemolysins including suilysin (SLY), plasminogen receptors, and arginine deiminase (*arc*A) [9].

Considering antimicrobial susceptibility of *S. suis* strains, recent studies have described increases in resistance rates to some antimicrobial classes. Resistance to lincosamides and macrolides has been increasing, both for pigs and human strains, and resistance to sulfonamides and tetracycline showed high prevalence [8]. Resistance to cephalosporin was already described in Europe and China, but resistance prevalence to penicillin, ampicillin, and ceftiofur remains low in most countries [8,10]. 

Emergence of multidrug resistant *S. suis* strains has also been described in humans and pigs, including asymptomatic animals, with highlight for the Asian epidemic clones [8]. Phenotypic and genetic studies suggest that swine may be reservoirs for the spread of antibiotic-resistant *S. suis* strains, which demands attention for the public health risk [8].

Brazil is a major producer and exporter of pork, occupying for several years the fourth place as producer and exporter in the world [4]. This position demands attention to swine health issues. Currently, special efforts are required to reduce antimicrobial usage and monitor resistance rates among bacterial pathogens that affect animals and humans. 

The study described here presents the characterization of an historic collection of *S. suis* isolated from diseased pigs from the most important pig producer states in Brazil, between 2001 and 2016. The strains were evaluated by pulsed-field gel electrophoresis (PFGE), molecular serotyping, PCR typing of virulence markers, and determination of resistance profile. The information about antimicrobial resistance of Brazilian strains during the 15-year period may reveal important changes in agent behavior, according to the increasing use of antimicrobials observed in the country in recent years.

## 2. Results

### 2.1. Molecular Serotyping

The primers described by Okura et al. [11] enabled identification of the following serotypes: 2/½, 3, 6, 7, 8, 1/14, 18, 27, and 28. Among these, serotype 2/½ was the most frequent (86.0%) followed by serotype 3, 7, and 1/14 (2.8% each). It is noteworthy that this protocol does not enable the differentiation of serotypes 2 from ½, and 1 from 14. Only six strains were classified as non-typeable. Regarding isolation sites, we observed that strains originating from the respiratory tract presented a higher diversity of serotypes, followed by the central nervous system, whereas in the other isolation sites, there was a predominance of serotype 2/½ (Table 1).

### 2.2. Virulence Profile Identification

Of the 215 *S. suis* strains, 78.1% were positive for the *sly* gene, 50.2% positive for *epf,* and 84.2% positive for *mrp* gene. The most frequent gene was *arcA* that was detected in 98.1% of the studied strains. Only four strains were negative for the detection of the assessed virulence genes. For the strains positive for *epf *and *mrp* gene detection, simple PCR reactions were performed to identify variant sequences as previously described [12]. However, no variants were identified for the *epf* gene, and all strains had amplification of a single fragment of ~ 744 bp. For the *mrp* gene, three variants were identified—*mrpV1*: 747 bp, *mrpV2*: 1148 bp, and *mrpV3*: 1556 bp. Among the strains positive for the *mrp* gene (181/215), the most frequent variant was *mrpV2* (137/181—75.7%), followed by *mrpV1* with 17.1% and *mrpV3* with only 7.2%.

From the screening of the four virulence genes studied and the *mrp* variants, 11 different virulence profiles were identified among the Brazilian *S. suis* strains (V1–V11, Table 2). Notably, the V2 profile (*sly+/arc*A*+/epf+/mrpV2*) was detected in 40.0% of the studied strains, followed by the V5 profile (*sly+/arc*A*+/epf-/mrpV2*) present in 17.7%, and the V6 (*sly-/arc*A*+/epf-/mrpV1*) detected in 10.2% of *S. suis* strains.

The evaluation of virulence profile distribution in relation to isolation sites (Table 3) resulted in significant statistical difference. Profiles V1, V2, V5, and V6 show a higher proportion in strains originating from the central nervous system, while V4, V8, V9, and V10 profiles appear to be more frequent in strains isolated from the respiratory system.

Regarding distribution of virulence profiles among identified serotypes, there is a bias of the large number of serotype 2/½ strains (86.0%). Nevertheless, the most frequent profile (V2) was more prevalent in *S. suis* serotype 2/½ strains. However, it can also be observed that profiles V6, V9, and V10 appear to be more frequent in other serotypes, such as 3 and 7, when compared to other virulence profiles.

### 2.3. Antimicrobial Resistance Profiling

High resistance rates to tetracyclines, macrolides, clindamycin, and sulfamethoxazole were identified, while the most effective antimicrobials were β-lactams, fluoroquinolones, tiamulin, and florfenicol. Aminoglycosides also stand out, since more than 30% of *S. suis* strains were resistant to gentamicin and neomycin, while 92.1% were susceptible to spectinomycin. Table 4 shows distributions of observed MIC values for the different tested antimicrobials and the respective MIC50 and MIC90. Sulfamethoxazole and the association trimethoprim-sulfamethoxazole were only tested in a single concentration each (256 and 2/38 µg/mL, respectively). 13.3% (34/256) of the strains were resistant to trimethoprim-sulfamethoxazole; this data could not be included in Table 4 due to the MICs range disposition.

**Figure 1:**
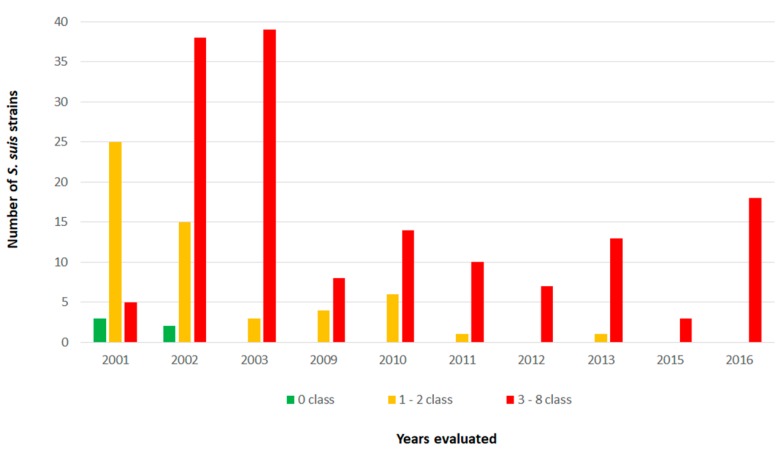
Distribution of studied *S. suis* strains according to isolation year and number of resistant antimicrobial classes.

Multidrug resistance was observed in 72.1% (155/215) of the evaluated strains using criteria described by Schwarz et al. [13] (Figure 1). The most susceptible strains were observed in higher proportion between 2001 and 2003, while the frequency of the most resistant strains started to grow from 2009 (more than six antimicrobial classes), with significant statistical difference (*p *< 0.001). Nevertheless, strains resistant to three to five antimicrobials classes are already present in high rates from 2001 to 2003, which indicates that, despite the isolation period, antimicrobial selection pressure was occurring.

**Figure 2:**
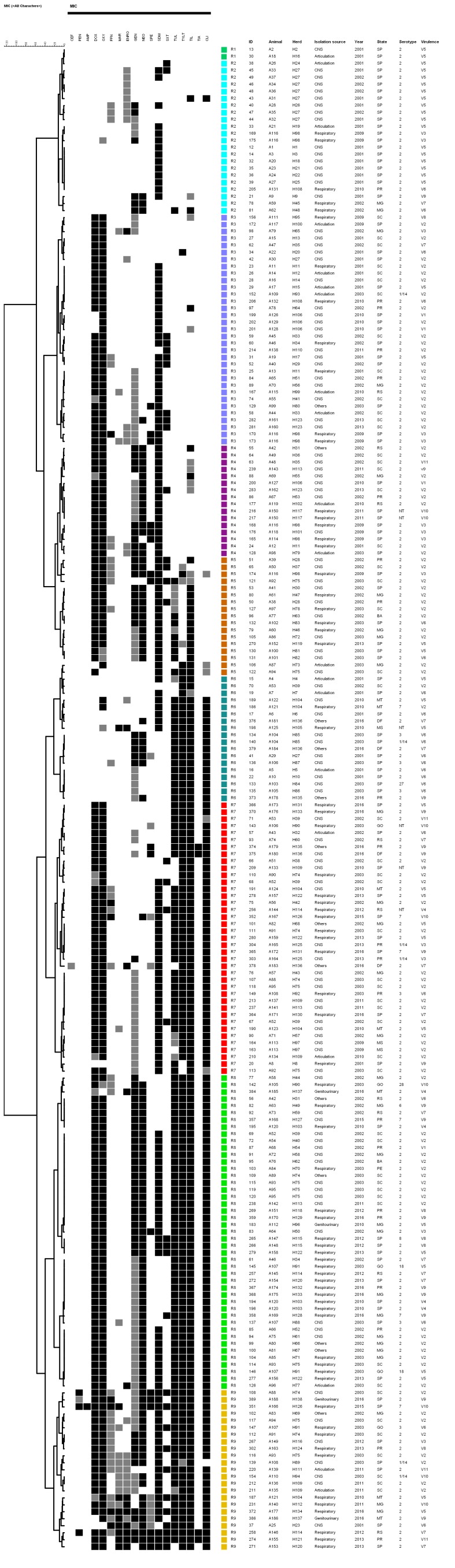
Resistance profile cluster analysis of Brazilian *S. suis* strains. The grey scale (black, grey, and white) corresponds to resistant, intermediate and sensitive status, respectively. The colored squares indicate the detected resistance profiles.

The resistance cluster analysis (Figure 2) enabled us to distinguish the studied strains into nine main groups (R1 to R9). The R1 and R2 clusters represent the strains with lower resistance and are composed of 24 strains isolated in 2001 and 2002. These less resistant strains were also characterized as a large proportion of the V5 virulence profile (18/24—75.0%). The R3 group comprises 33 strains isolated between 2001 and 2010, in which the virulence profile V2 is more frequent (21/33—63.6%). However, no significant increase in resistance was observed among these isolates, and only five multidrug-resistant strains were detected in this group. The R4 group, on the other hand, consists of 16 multidrug-resistant strains, characterized by their resistance to tetracyclines, aminoglycosides, and sulfonamides. This same resistance pattern is maintained in the R5 group that still has macrolide resistance. Similarly, the cluster is composed of 17 multidrug-resistant strains isolated mostly (15/17—88.2%) between 2002 and 2003.

The R7 group is composed of 39 strains resistant to tetracyclines, sulfonamides, macrolides, and lincosamides. The R8 and R9 clusters are composed of the most resistant strains, especially R9, which contains strains resistant to six to eight antimicrobial classes. These groups are characterized at the least by resistance to tetracyclines, aminoglycosides, sulfonamides, macrolides, and lincosamides. Although there is no direct relationship between resistance profiles, origins, and serotypes of the studied strains, it is significant that 72.0% (18/25) of the isolates originated between 2015 and 2016 comprise the R7, R8, and R9 resistance clusters.

### 2.4. Pulsed-Field Electrophoresis Analysis

**Figure 3:**
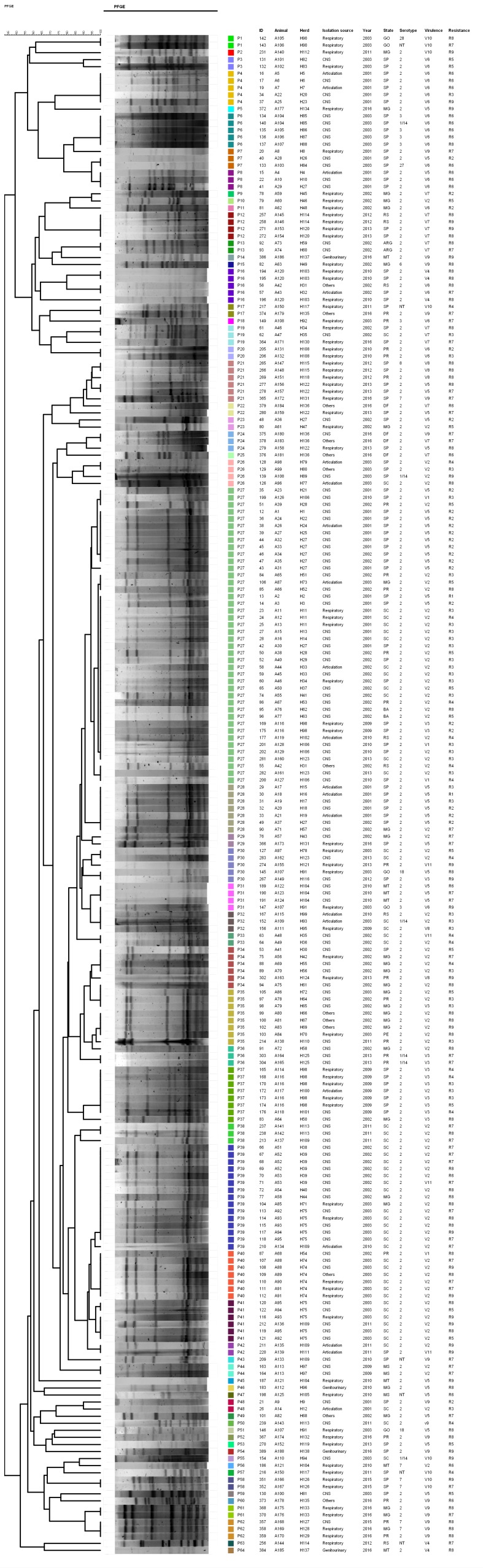
Dendrogram showing the relationship among pulsed-field gel electrophoresis (PFGE) restriction profiles of Brazilian *S. suis* strains.

In the PFGE cluster analysis, 64 pulsotypes (P1–P64) were identified. In the dendrogram (Figure 3), two main groups can be distinguished: The first comprises 20 pulsotypes (P1–P20) with high heterogeneity between the restriction profiles when compared to the second group, which is composed of 161 strains clustered into 42 pulsotypes (P23–P64) that show greater homogeneity between restriction profiles. 

Among the 64 pulsotypes, there is a tendency to group strains according to year of isolation. However, no association was detected among the pulsotypes and the sample origin, isolation site, and antimicrobial resistance profile. Regarding serotypes, it is notable that 37 pulsotypes, among them the largest P27, P28, P35, P37, and P39, are composed of serotype 2/½ strains. Only eight pulsotypes were solely formed by other serotypes besides 2/½.

Regarding the association between genotypes and virulence profiles, we observed that the pulsotypes P3, P4, P6, and P20 are comprised entirely by strains characterized as V6, the second largest virulence profile identified. Similarly, the strains that compose the largest virulence profile (V2) are distributed in a large proportion among the largest PFGE genotypes—P27 (25/42), P34 (5/6), P35 (7/8), P39 (14/15), and P40 (5/6).

## 3. Discussion

*Streptococcus suis* is one of the pathogens with the greatest impact on worldwide pig production [14]. In addition, this pathogen has zoonotic potential for those working directly or indirectly in pig production [15]. Thus, the phenotypic and genotypic characterization of the agent is extremely important to understand its epidemiology and control. 

In Brazil, serotype 2 is described as the most prevalent, followed by serotypes ½, 14, 7, and 9 [16,17,18,19]. The results of molecular serotyping in the present study corroborate the literature with a predominance of serotype 2/½, which was identified in 86.0% (185/215) of strains from all sites and years of isolation. In addition, eight more serotypes were detected (3, 6, 7, 8, 18, 27, 28, and 1/14). To our knowledge, this is the first report of *S. suis* serotype 27 in Brazil. Only 2.8% of the studied strains were characterized as non-typeable by molecular serotyping. In their study, Okura et al. [11] described more than 15% of *S. suis* strains as non-typeable by both conventional (co-agglutination) and molecular serotyping. 

According to the previously published pathotype category system [20,21], the studied Brazilian *S. suis* strains were considered either “opportunistic”—originated from lung of pigs with pneumonia—or “pathogenic” invasive strains. Previous studies in Europe and Asia reported that the presence of three virulence genes (*sly*, *epf*, *mrp*) is associated with more virulent strains from the most prevalent serotypes [22,23]. In the present study, 47.9% of the strains were positive for such genes and 95.1% (98/103) of these belong to serotype 2/½. 

Despite the greater number of “opportunistic” strains, a positive association was observed between some virulence profiles and isolation sites, in which invasive strains isolated from the central nervous system (CNS) were characterized as profiles V1, V2, V5, and V6. Only 12 strains were negative for the *sly*, *epf,* and *mrp* genes, of which eight were positive for the *arc*A gene. Among these strains, 75.0% (9/12) were isolated from the respiratory tract, corroborating literature that affirms that less virulent profiles are related to noninvasive infections [21].

However, three of these apparently less virulent strains were also characterized as invasive, originating from the CNS and joint. Previous studies have reported low virulence profiles in invasive strains. Gottschalk et al. [24] described 77.0% of invasive strains with an MRP-EF- virulence profile, while Zheng et al. [25] detected 78.5% of the Canadian strains with *mrp*−/*sly*−/*epf*− profile and no apparent relation with their serotypes and origin was identified.

Meanwhile, the *arc*A gene was detected in 98.6% (212/215) of the studied strains. Arginine deiminase, encoded by the *arc*A gene, is described as a facilitating factor for *S. suis* survival under acidic conditions [26]. Previous studies have also identified a high frequency of this gene in invasive and respiratory *S. suis* strains isolated from swine and humans [27,28], which suggests that it is not a good virulence marker.

Regarding antimicrobial resistance profiling, the high resistance rates to tetracyclines, macrolides, clindamycin, and sulfamethoxazole detected in the present study appear to be common in *S. suis* in recent years, since they are present in several studies carried out in different geographic areas [8,10,29]. In addition, a very low rate of β-lactams resistance was observed among the Brazilian *S. suis* strains, regardless of their origin. β-lactam susceptibility is often reported in *S. suis*. Hernandez-Garcia et al. [10] and Zhang et al. [30] reported less than 5.0% resistance to penicillin and cephalosporins among British and Chinese strains. Yongkiettrakul et al. [8] also reported low β-lactams resistance in Thai strains isolated from 2012–2015. Considering these reports, it has been suggested that the β-lactams, as well as florfenicol, are still good options for treatment of *S. suis* infections in Brazil.

In the present study, an association tendency between multidrug-resistant strains and isolation years was identified. We observed that most strains with higher susceptibility (48/60–80.0%) were isolated from 2001–2003, while the more resistant strains (resistance to six and up to eight classes) were isolated from 2013–2016. Yongkiettrakul et al. [8] reported similar results when comparing strains isolated in Thailand from 2006–2007 and 2012–2015, describing an increase in resistance for some antimicrobials such as clindamycin, doxycycline, erythromycin, and tetracycline. Hernandez-Garcia et al. [10] also compared strains of healthy and sick pigs over two periods, from 2009–2011 and 2013–2014, and described similar results for the same antibiotics.

Regarding the PFGE analysis, we only observed a clustering trend of strains according to the isolation year and a slight association between some virulence profiles and pulsotypes, especially for serotype 2/½ strains. However, no direct association was observed within genotypes and strains isolation sites and origin data, which also corroborates the literature. The temporal clustering may be due to the genetic changes of *S. suis* during the different periods from which these strains originated. Nevertheless, no clonal dispersion or persistency was detected over the analyzed years and herds. Previously, Mai et al. [31] reported that human *S. suis* strains also grouped by isolation year. Similarly, Tharavichitkul et al. [32] and Huang et al. [33] described the tendency to cluster pulsotypes with virulence profiles, which was also observed in *S. suis* strains isolated from rabbits [34].

The high heterogeneity of *S. suis* PFGE restriction profiles was also previously described. For *S. suis* specifically, a variability of five to 13 bands was described in restriction profiles using the SmaI enzyme [35,36]. This heterogeneity of genotypes identified in several studies demonstrates a high genetic variability in the strains circulating in different regions of Brazil.

The results obtained showed that *S. suis* is an important pathogen with large genetic diversity and a rising tendency for future antimicrobial resistance problems, since the multidrug resistance strains were identified in large rates. Evaluation of genetic and resistance profiles must become more common and frequent in a large country such as Brazil, in order to permit and guide decisions on appropriate antibiotic use and vaccine prevention of *S. suis* infections in the pig industry. The human healthcare system must be also subject of constant monitoring to evaluate whether the pathogen becomes a zoonotic risk in the region, where, until now, there are no cases registered of human infection by this agent.

## 4. Materials and Methods

### 4.1. Bacterial Strains

In the present study, 215 strains of *Streptococcus suis* were isolated from pigs with clinical signs of encephalitis, septicemia, arthritis, or pneumonia from the states of São Paulo, Santa Catarina, Paraná, Pernambuco, Bahia, Minas Gerais, Goiás, and Rio Grande do Sul, from 2001–2016. The clinical samples were received, and the isolated strains were stored at −86 °C at the Laboratory of Swine Health from the Faculdade de Medicina Veterinária e Zootecnia—USP. This study was approved by the Ethics Committee under protocol number 1216220316.

Strains were grouped according to the *S. suis* isolation site: Respiratory system (33.5%—72/215), central nervous system (CNS) (49.3%—106/215), genitourinary system (1.9%—4/215), joints (9.3%—20/215), and other sites, including samples from spleen, heart, liver, pericardium, peritoneum, and blood, that present in most cases animals with septicemia (6.0%—13/215). Among the strains isolated from the respiratory system, only two originated from the nasal cavity, while the remaining strains were isolated from the lungs of animals showing signs of pneumonia.

### 4.2. Bacterial Reactivation

The samples were plated on sheep blood agar (5%), MacConkey agar, and chocolate agar (Difco-BBL, Detroit, MI, USA), and incubated at 37 °C for 24 h. Suspect *S. suis* isolates, which showed alpha hemolysis were subcultured onto fresh plates for isolation and the selected strains were stored at −86 °C until complete identification and further analysis.

### 4.3. Identification by MALDI-TOF Mass Spectrometry

The identification of the protein spectra of strains was performed by matrix assisted laser desorption ionization time of flight mass spectrometry (MALDI-TOF MS) using the protocol previously described by Hijazin et al. [37]. Bacterial mass spectra in the range of 2–20 kDa were acquired using a Microflex™ mass spectrometer (Bruker Daltonik, Bremen, Germany) and identified with the MALDI BioTyper™ 3.0 software. Each sample was applied to two wells of the plate and two readings were taken for each sample. The obtained spectra were compared to the manufacturer’s library and the standard Bruker interpretative criteria were applied: Scores between ≥1.7 and <2.0 were only accepted for genus identification and scores ≥2.0 were accepted for species identification.

### 4.4. DNA Extraction

DNA extraction was performed with Boom et al. [38] protocol with initial lysozyme (100 mg/mL) and proteinase K (20 mg/mL) (US Biological, Swampscott, MA, USA) digestion at 37 ° C for 60 min. Samples were stored at – 20 °C for further PCR serotyping and virulence profiling.

### 4.5. Molecular Serotyping

Serotype identification was performed by multiplex PCR using primers described by Okura et al. [11]. The multiplex PCR protocol was adapted by performing duplex reactions to avoid misidentification of bands of similar sizes. 

### 4.6. Virulence Gene Identification

Four genes associated with *S. suis* virulence were assessed: *sly*—suilysin, *arc*A—arginine deiminase, *epf*—extracellular factor, and *mrp*—muramidase-released protein. For the identification of *sly*,* epf*, *mrp* genes and variants of *epf* and *mrp*, Silva et al. [12] primers were used; and for the *arc*A gene, the ones described by Maneerat et al. (2013) [27].

### 4.7. Amplification and Electrophoresis

PCR reactions were done with 50 μL in total, of which 5 μL were genomic DNA, and the remaining composed of ultrapure water, 10 × PCR buffer, 1.5 μM MgCl, 200 μM dNTPs, 200 μM of each primer, and 1.25 U of Taq polymerase. Electrophoresis was performed using a 1.5% agarose gel. The fragments were stained with BlueGreen ™ (LGC Biotecnologia, São Paulo, Brazil), visualized in a Gel Doc XR photo documentation system (Bio-Rad Laboratories, Hercules, California, USA) and compared to 100 bp DNA ladder (New England BioLabs Inc., Ipswich, MA, USA).

### 4.8. Pulsed-Field Gel Electrophoresis

The preparation of bacterial DNA for molecular typing by PFGE technique was performed as described by Vela et al. [36], followed by an enzymatic restriction with SmaI (New England BioLabs Inc., Ipswich, MA, USA) and incubation at 25 °C for 20 h. The electrophoresis was performed for 18 h at 6 V/cm, 120° fixed angle, with initial pulse of 0.5 s and final of 40 s in a 0.5X Tris-borate-EDTA buffer kept at 14 °C. The gel was stained in a SYBR ™ Safe solution (Thermo Fisher Scientific, Rockford, IL, USA) for 30 min and photographed by the Doc XR Gel System (Bio-Rad Laboratories, Hercules, CA, USA); fragments were identified based on the high molecular weight CHEF DNA size Standard-lambda ladder marker (Bio-Rad Laboratories, Hercules, CA, USA).

### 4.9. Antimicrobial Resistance Profiling

For the identification of antimicrobial resistance profiles, the broth microdilution technique was used according to the standards defined in the CLSI document VET01, fifth edition [39] to determine the minimum inhibitory concentrations (MICs). For this purpose, a microplate panel was assembled composed of the following antimicrobials (MilliporeSigma, St. Louis, MO, USA): Ampicillin, ceftiofur, penicillin, doxycycline, oxytetracycline, marbofloxacin, enrofloxacin, florfenicol, espectinomycin, gentamycin, neomycin, trimethoprim-sulfamethoxazole, sulfamethoxazole, clindamycin, tylosin, tulathromycin, tilmicosin, and tiamulin. 

*Streptococcus pneumoniae* ATCC 49619 and *Staphylococcus aureus* ATCC 29213 were used as quality control strains. 

MIC results were categorized as susceptible, intermediate, and resistant using the clinical interpretative criteria specified in CLSI performance standard VET08, fourth edition [40]. If interpretive criteria were not present in VET08, fourth edition [40], CLSI performance standard M100, twenty-eighth edition, was used [41]. The determination of MIC50, MIC90, and the multidrug resistance rate were performed as described by Schwarz et al. [13].

### 4.10. Statistical Analysis

The frequency distribution of resistance and virulence profiles of the studied strains according to origin and serotype were performed using the SPSS 16.0 (SPSS Inc). The profiles and results of resistance and virulence genes detection were treated as categorical variables and the differences analyzed by Fisher-Freeman-Halton test with bilateral probability estimated by Monte Carlo method and 5% significance level.

For the genotype cluster analysis, the BioNumerics 7.6 (Applied Maths NV, Sint-Martens-Latem, Belgium) software was used. A dendrogram was constructed using the Dice coefficient and the UPGMA (unweighted pair group method with arithmetic mean) method; the distinction of pulsotypes was determined by at least four bands of difference between strains [42].

The resistance results were also transformed into binary data for identification of the respective resistance profiles and subsequent cluster analysis. Profiles were analyzed as categorical data in BioNumerics 7.6 (Applied Maths NV, Sint-Martens-Latem, Belgium), and a dendrogram was constructed using the different values coefficient and Ward method.

## 5. Conclusions

This temporal evaluation of *S. suis* strains of different states of Brazil could reveal a large diversity of these pathogens considering resistance patterns, virulence genes, and PFGE profiles. In addition to the current extensive use of antimicrobials in pig production to prevent and control bacterial diseases in the country, most *S. suis* strains isolated are still susceptible to beta-lactam antibiotics and florfenicol. On the other hand, the multidrug resistance of *S. suis* strains represents a high percentage of studied isolates, demonstrating that pigs must be carefully considered as reservoirs for the spread of antibiotic-resistance genes. This also demands attention for the risk of multidrug resistance emergence for the public health. 

## Figures and Tables

**Table 1:** Distribution of the studied strains according to isolation site and serotype—N (%).

Serotype	Isolation Site					Total
	Joints	Genitourinary System	Other Sites	Respiratory	CNS	
**2/½**	19 (95.2)	4 (100)	13 (100)	55 (76.3)	94 (88.6)	185 (86)
**3**	0	0	0	2 (2.9)	4 (3.7)	6 (2.8)
**7**	0	0	0	5 (6.9)	1 (0.9)	6 (2.8)
**1/14**	1 (4.8)	0	0	0	5 (4.6)	6 (2.8)
**NT**	0	0	0	5 (6.9)	1 (0.9)	6 (2.8)
**18**	0	0	0	2 (2.9)	0	2 (0.9)
**27**	0	0	0	0	1 (0.9)	1 (0.5)
**28**	0	0	0	1 (1.5)	0	1 (0.5)
**6**	0	0	0	1 (1.5)	0	1 (0.5)
**8**	0	0	0	1 (1.5)	0	1 (0.5)
**Total**	20 (100)	4 (100)	13 (100)	72 (100)	106 (100)	215 (100)

CNS: central nervous system.

**Table 2:** Virulence profiles identified in the *Streptococcus suis* strains studied, considering the muramidase-released protein (*mrp)* gene variations—N (%).

Profile	Virulence Genes	Frequency
**V1**	*sly+/arc*A*+/epf+/mrpV1*	4 (1.9)
**V2**	*sly+/arc*A*+/epf+/mrpV2*	86 (40.0)
**V3**	*sly+/arc*A*+/epf+/mrpV3*	13 (6.0)
**V4**	*sly+/arc*A*+/epf-/mrpV1*	5 (2.3)
**V5**	*sly+/arc*A*+/epf-/mrpV2*	38 (17.7)
**V6**	*sly-/arc*A*+/epf-/mrpV1*	22 (10.2)
**V7**	*sly-/arc*A*+/epf-/mrpV2*	13 (6.0)
**V8**	*sly+/arc*A*+/epf+/mrp-*	5 (2.3)
**V9**	*sly+/arc*A*+/epf-/mrp-*	17 (7.9)
**V10**	*sly-/arc*A*+/epf-/mrp-*	8 (3.7)
**V11**	*sly-/arc*A*-/epf-/mrp-*	4 (1.9)

**Table 3:** Distribution of *S. suis* virulence profiles according to isolation site—N (%).

Profile	Isolation Site					Total
	Joints	Genitourinary System	Other Sites	Respiratory System	CNS	
**V1**	0	0	0	0	4 (100)	4 (100)
**V2**	11 (12.8)	0	6 (7.0)	16 (18.6)	53 (61.6)	86 (100)
**V3**	0 (0)	0	0	7 (53.8)	6 (46.2)	13 (100)
**V4**	0	1 (20.0)	0	4 (80.0)	0	5 (100)
**V5**	5 (13.2)	1 (2.6)	1 (2.6)	11 (28.9)	20 (52.6)	38 (100)
**V6**	3 (13.6)	0	1 (4.5)	6 (27.3)	12 (54.5)	22 (100)
**V7**	0	0	3 (23.1)	7 (53.8)	3 (23.1)	13 (100)
**V8**	0	0	0	5 (100)	0	5 (100)
**V9**	0	2 (11.8)	2 (11.8)	8 (47.1)	5 (29.4)	17 (100)
**V10**	0	0	0	7 (87.5)	1 (12.5)	8 (100)
**V11**	1 (25.0)	0	0	1 (25.0)	2 (50.0)	4 (100)

CNS: Central nervous system. Estimated probability by Fisher-Freeman-Halton test *p* < 0.001.

**Table 4:** Minimum inhibitory concentration (MIC) values distribution, MIC50 and MIC90 values, and resistance rates of studied *S. suis* strains against tested antimicrobials.

Antimicrobial	MIC Range (µg/mL) ^1^														MIC50 (µg/mL)	MIC90 (µg/mL)	Resistance N (%)
	0.06	0.12	0.25	0.5	1	2	4	8	16	32	64	128	256	512			
**Ampicillin**	0	0	214	0	0	1	0	0	0	0	0	0	0	0	0.25	0.25	1 (0.5)
**Ceftiofur**	0	0	212	0	0	1	2	0	0	0	0	0	0	0	0.25	0.25	0
**Penicillin**	0	209	3	0	3	0	0	0	0	0	0	0	0	0	0.12	0.12	3 (1.4)
**Doxycycline**	0	0	0	49	7	20	45	69	25	0	0	0	0	0	4.0	>16.0	159 (74.0)
**Oxytetracycline**	0	0	0	42	5	7	22	30	109	0	0	0	0	0	>16.0	>16.0	168 (78.1)
**Marbofloxacin**	3	5	13	79	93	13	1	8	0	0	0	0	0	0	1.0	2.0	9 (4.2)
**Enrofloxacin**	0	17	72	91	24	3	0	8	0	0	0	0	0	0	0.5	1.0	12 (5.6)
**Florfenicol**	0	0	3	8	64	99	34	5	2	0	0	0	0	0	2.0	4.0	7 (3.3)
**Spectinomycin**	0	0	0	0	0	0	0	73	88	28	9	3	14	14	16.0	64.0	17 (7.9)
**Gentamicin**	0	0	0	0	31	39	80	52	12	1	0	0	0	0	4.0	8.0	65 (30.2)
**Neomycin**	0	0	0	0	0	0	68	41	47	37	22	0	0	0	8.0	>64.0	106 (49.3)
**Clindamycin**	0	0	88	2	1	4	2	7	111	0	0	0	0	0	>16.0	>16.0	120 (55.8)
**Tylosin**	0	0	0	73	11	4	0	4	7	6	110	0	0	0	>64.0	>64.0	126 (58.6)
**Tilmicosin**	0	0	0	0	0	0	41	21	10	13	11	119	0	0	>128.0	>128.0	143 (66.5)
**Tulathromycin**	0	0	0	0	57	9	8	6	5	9	7	113	0	0	>128.0	>128.0	120 (55.8)
**Tiamulin**	0	0	0	122	33	31	11	8	6	3	1	0	0	0	0.5	4.0	4 (1.9)
**Sulfamethoxazole**	0	0	0	0	0	0	0	0	0	0	0	59	0	156	512	512	156 (60.9)

^1^ Number of strains with the MIC value for the respective antimicrobials. Respective antimicrobials range tested are contained in the white area. Resistance breakpoints are indicated with the thick black vertical lines for each tested antimicrobial.
